# Correction: Effects of nitrogen, phosphorus and potassium formula fertilization on the yield and berry quality of blueberry

**DOI:** 10.1371/journal.pone.0318032

**Published:** 2025-01-17

**Authors:** Xinyu Zhang, Shuangshuang Li, Xiaoli An, Zejun Song, Yunzheng Zhu, Yi Tan, Xiaolan Guo, Delu Wang

There is an error in affiliation 3 for author Xiaolan Guo. The correct affiliation 3 is: College of Life Sciences, Huizhou University, Huizhou, Guangdong, China.

In the Abstract section, there is an error in the fifth sentence. The correct sentence is: Based on the comprehensive evaluation of principal component analysis and multi factor analysis of variance, the best fertilization combination for high-yield and good-quality blueberries was N1P2K2 (F2), that is, the best fertilization effect was that including N 100 g/plant, P_2_O_5_ 25 g/plant, K_2_O 50 g/plant, applied in the form of ammonium sulfate (472 g/plant), superphosphate (41 g/plant) and potassium sulfate (79 g/plant), respectively.

In the Effects of different formulations of fertilization on blueberry fruit yield subsection of Results, there is an error in the fourth sentence. The correct sentence is: The most effective was F2 treatment, which was 79.26% higher than CK. Range analysis showed that N2P3K2 and N3P2K2 were the best fertilizer combinations affecting the fruit weight and yield per plant of blueberry.

In the Effect of different formula fertilization on blueberry fruit quality subsection of Results, there is an error in the first sentence of the fifth paragraph. The correct sentence is: The range analysis showed that the optimal combination affecting the content of anthocyanins, total phenols, flavonoids, soluble protein, soluble solids, soluble sugar, titratable acidity and sugar acid ratio of blueberry fruits were N2P1K3, N3P3K1, N1P1K2, N3P3K1, N1P1K2 or N2P1K2, N3P2K2, N2P3K1 and N1P2K2, respectively.

In the Conclusion, there is an error in the fifth sentence of the first paragraph. The correct sentence is: Therefore, through comprehensive evaluation and variance analysis, this study concluded that the best combination of N, P and K fertilization formula for blueberry cultivation with high yield, quality and efficiency in this area was N1P2K2 (F2), that is, 100, 25 and 50 g/plant of N, P_2_O_5_ and K_2_O, visually.
