# Supplementary material for: Effects of nitrogen, phosphorus and potassium formula fertilization on the yield and berry quality of blueberry
Source: PLoS One. 2023 Mar 16;18(3):e0283137. doi: 10.1371/journal.pone.0283137 (PMC10019695; doi:10.1371/journal.pone.0283137)
Supplement: Attachment — Submitted filename: rebuttal letter.docx [file pone.0283137.s001.docx]

**Detailed Response to Reviewers**

**Manuscript Number:** PONE-D-22-30089

**Title:** Effects of nitrogen, phosphorus and potassium formula fertilization on the yield and quality of blueberry fruit

**Authors:** Xinyu Zhang^1^,Shuangshuang Li^2^, Delu Wang^1*^,Xiaoli An^1^,Zejun Song^1^, Yunzheng Zhu^1^,Yi Tan^1^,Xiaolan Guo^3^

Dear Editor: Sushanta Kumar Naik and Dear reviewers:

Thank you for your useful comments on our manuscript. We wish to give a sincere gratitude to referees for reviewing our paper carefully. We apologize for any inconveniences caused by these errors. We have modified the manuscript accordingly, and the response to the referees’ comments are listed point by point below:

**Additional Editor Comments:**

**Comments:** Give justification that how a one-year trial conducting at a single experimental site is suitable to improve the production of blueberry outside that site.Several experiments need to be conducted across different agroecosystems due to varying yield-impacting factors to conclude for its recommendation. How is your present investigation justify the fertilizer recommendation across the region?

**Reply:** Thanks for your comment. This research has been conducted for three years, so I re discussed this issue in the results and analysis, added the data results of 2016, and proved that F2 is indeed better than F4, which was revised in the manuscript. In addition, the research is based on the preliminary literature review, pre experiment and local fertilization experience of our research group, so it can be said that the fertilization suggestions for the whole region are relatively reasonable. We also measured the soil background values (pH value is 4.35~5.50, organic matter content is 23.90 g/kg, total N content is 0.784 g/kg, total P content is 0.19 g/kg, and total K content is 2.8 g/kg) before the test treatment. We believe that the proportion of N, P, K fertilizers used can be appropriately adjusted according to the content of N, P, K in the soil of different regions to improve the blueberry yield outside the site.

**Comments:** Re-write the entire manuscript based on proper English usage or consult to a professional.

**Reply:** Thanks for your suggestion. The manuscript has been revised according to correct English usage.

**Reviewer #1:** This is a good attempt to show the importance of major nutrients in production and quality. However, some important aspects are missing in this manuscript which is mentioned below;

**Comment:** What is the basis of selection of three levels of N, P and K (based upon the recommendation like optimum, sub-optimum or soil test values etc.), as no information was provided on the soil properties.

**Reply:** Thanks for your comment. Information on soil properties is provided in the test site profiles section in lines 95-97. The three levels of N, P and K were selected based on the previous literature review of our research group (such as Guo et al. (2021) to explore the effects of water and fertilizer coupling on the physiological characteristics and growth of rabbiteye blueberry), preliminary experiments and local fertilization experience.

**Comment:** Beside the primary nutrients, micro nutrient affects the quality very significantly, but nothing was informed in this aspect.

**Reply:** Thanks for your comment. This study mainly focused on the effect of N, P and K on the yield and quality of blueberry, so it did not take this into account.

**Comment:** The experiment was carried out in 2014, the result presented is based on one year data or else.

**Reply:** Thanks for your comment. The test results are based on the pre experiment and two-year test data, and the relevant content has been added to lines 207-212 of the manuscript.

**Comment:** The work was related with the application of N, P and K on yield and quality, but use/ recommendation of these fertilizer only, may create the imbalance of micro nutrient and other soil properties. Which is an important aspect that is missing.

**Reply:** Thanks for your comment. This study focused on the impact of N, P and K on the yield and quality of blueberry, so it did not pay attention to trace elements and determine their content changes.

**Comment:** Based upon comprehensive score, it was recommended that F2 is superior but F4 and F9 is second and third, F2 and F4 were very similar. So it is better to compare the benefit cost ratio also before recommendation.

**Reply:** Thanks for your comment. Related comparisons have been added on lines 212-213. the fertilization cost of treatment F2 and F4 is $2.278 and $2.281 respectively, and the yield of treatment F2 is higher than that of treatment F4. Therefore, the benefit cost ratio of F2 treatment is greater than that of F4, so F2 treatment is the best.

**Reviewer #2:** In general, the manuscript needs extensive revision for English language and grammar by a professional.

**Reply:** Thanks for your suggestion. The manuscript has been revised according to correct English usage.

**Comment:** Page 1, title: … and potassium fertilization formula on the yield and berry quality of blueberry

**Reply:** Thanks for your suggestion. The title has been modified in the manuscript.

**Comment:** Page 2, line 21: …of nitrogen (N), phosphorus (P) and potassium (P) in the field…From this line and throughout the rest text you must use the above abbreviations for the three nutrients.

**Reply:** Thanks for your suggestion. Relevant contents have been modified in the manuscript.

**Comment:** Page 2, line 23: blueberry fruits, aiming to optimize the amount of supplied fertilizers.

**Reply:** Thanks for your suggestion. Relevant contents have been modified in lines 23-24 of the manuscript.

**Comment:** Page 2, line 28: Even the treatment with the worst results (N2P3K1), …

**Reply:** Thanks for your suggestion. It has been revised in line 28 of the manuscript.

**Comment:** Page 2, line 30: times higher than the control, respectively.

**Reply:** Thanks for your suggestion. Relevant contents have been modified in lines 30-31 of the manuscript.

**Comment:** Page 2, line 33: blueberries was that including N 100 g/plant, P_2_O_5_ 25 g/plant, K_2_O 25 g/plant, applied in the form of ammonium sulfate (472 g/plant), superphosphate (41 g/plant) and potassium sulfate (40 g/plant), respectively.

**Reply:** Thanks for your suggestion. Relevant contents have been modified in lines 33-36 of the manuscript.

**Comment:** Page 3, line 41: potassium are well-known mineral … development [1], and the application of fertilizers containing these elements can significantly improve the yield…

**Reply:** Thanks for your suggestion. Relevant contents have been modified in lines 41-43 of the manuscript.

**Comment:** Page 3, line 43-51: These lines must be written better.

**Reply:** Thanks for your suggestion. The sentences mentioned have been revised in lines 44-52 of the manuscript.

**Comment:** Page 3, line 58: but the yield per unit area and quality are not

**Reply:** Thanks for your suggestion. It has been revised in line 59 of the manuscript.

**Comment:** Page 4, line 65: “…they often have the problem …potassium fertilizer.” What do you mean?

**Reply:** Thanks for your suggestion. What I mean here is that blueberries belong to shallow root oligotrophic plants(Aimé et al., 2017; Medvecký et al., 2021), they prefer ammonium N (Douglas et al., 2017), and have low demand for P, K, Ca, and Mg (Wu et al., 2019), there is often a phenomenon of more application of N fertilizer and less application of P and K fertilizer. Relevant contents have been modified in lines 64-66 of the manuscript.

**Comment:** Page 4, line 68: fertilization affect negatively the yield

**Reply:** Thanks for your suggestion. It has been revised in line 68 of the manuscript.

**Comment:** Page 4, line 69: and the vegetative growth of blueberries is inhibited, or even damaged [17].

**Reply:** Thanks for your suggestion. Relevant contents have been modified in lines 69-70 of the manuscript.

**Comment:** Page 4, line 71: cannot

**Reply:** Thanks for your suggestion. It has been revised in line 71 of the manuscript.

**Comment:** Page 4, line 72: but also it can improve their fruit…

**Reply:** Thanks for your suggestion. It has been revised in line 72 of the manuscript.

**Comment:** Page 4, line 76: have pointed out that applying conventional fertilizers without guidance, can lead to salt accumulation in soil and reduce…

**Reply:** Thanks for your suggestion. Relevant contents have been modified in lines 76-77 of the manuscript.

**Comment:** Page 4, line 77-78: Delete ‘by applying … habits [8].’

**Reply:** Thanks for your suggestion. The mentioned sentence has been deleted.

**Comment:** Page 4, line 80: single-nutrient fertilizer

**Reply:** Thanks for your suggestion. It has been revised in line 80 of the manuscript.

**Comment:** Page 5, line 83: rabbiteye blueberry cv. Brilliant plants, explores

**Reply:** Thanks for your suggestion. It has been revised in line 82 of the manuscript.

**Comment:** Page 5, line 85: test, aiming to optimize the

**Reply:** Thanks for your suggestion. It has been revised in line 84 of the manuscript.

**Comment:** Page 5, line 90: 2.1 Experimental site

**Reply:** Thanks for your suggestion. It has been revised in line 89 of the manuscript.

**Comment:** Page 5, line 91: Please add the geographic coordinates of this site

**Reply:** Thanks for your suggestion. The geographic coordinates of this site have been added in lines 91-92 of the manuscript.

**Comment:** Page 5, line 92: which is characterized by subtropical

**Reply:** Thanks for your suggestion. It has been revised in line 92 of the manuscript.

**Comment:** Page 5, line 95: Delete the second ‘soil’

**Reply:** Thanks for your suggestion. The mentioned sentence has been revised.

**Comment:** Page 5, line 96-97: Express these values as per cent (%)

**Reply:** Thanks for your suggestion. Relevant contents have been modified in lines 96-97 of the manuscript.

**Comment:** Page 5, line 98: 2.2 Plant material

**Reply:** Thanks for your suggestion. It has been revised in line 98 of the manuscript.

**Comment:** Refer whether these plants were self-rooted or grafted. Were the trees trained with a single trunk or what?

**Reply:** Thanks for your comment. All these plants are tissue cultured.

**Comment:** Page 5, line 99: Five -year-old rabbiteye blueberry (Vaccinium ??? cv. Brightwell) plants

**Reply:** Thanks for your suggestion. It has been revised in line 99 of the manuscript.

**Comment:** Page 5, line 101: 2.3 Experimental design

**Reply:** Thanks for your suggestion. It has been revised in line 101 of the manuscript.

**Comment:** Refer the plantation distances among trees.

**Reply:** Thanks for your suggestion. the plantation distances among trees has been added in lines 105 of the manuscript.

**Comment:** Page 5, line 102: …under field conditions. Fertilizers containing N, P and K doses at three doses for each nutrient, three …

**Reply:** Thanks for your suggestion. Relevant contents have been modified in lines 101-104 of the manuscript. .

**Comment:** Page 6, line 104: Non fertilized plants were used …

**Reply:** Thanks for your suggestion. It has been revised in line 105 of the manuscript.

**Comment:** Page 6, line 105: The fertilizers used were…

**Reply:** Thanks for your suggestion. It has been revised in line 106 of the manuscript.

**Comment:** Page 6, line 106-107: Delete the words ‘content’

**Reply:** Thanks for your suggestion. This word has been deleted from the manuscript.

**Comment:** Page 6, line 107: …63.2%), all of high chemical purity.

**Reply:** Thanks for your suggestion. This sentence has been revised in line 108 of the manuscript.

**Comment:** Page 6, line 109: Do you mean ‘fruit production’ or ‘fruit set’?

**Reply:** Thanks for your comment. Line 109 in the manuscript I mean “fruit production”.

**Comment:** Page 6, line 110: What do you mean with ‘reducing fertilizer’?

**Reply:** Thanks for your comment. It refers to reductive fertilizer, reducing is a noun, which means " reductive ". The relevant content has been modified in line 111 of the manuscript.

**Comment:** Page 6, line 110-111: The fertilizers were applied into a trench

**Reply:** Thanks for your suggestion. It has been revised in line 111 of the manuscript.

**Comment:** Page 6, line 112-113: canopy projection, mixed and covered with soil.

**Reply:** Thanks for your suggestion. It has been revised in line 113 of the manuscript.

**Comment:** Page 6, line 113: What you mean with ‘carried out’?

**Reply:** Thanks for your comment. This sentence means that the experiment started in 2014, and the relevant contents have been modified in lines 1113-114 of the manuscript.

**Comment:** Page 6, line 114: the experimental design is shown

**Reply:** Thanks for your suggestion. It has been revised in line 114 of the manuscript.

**Comment:** Page 7, Table 2: There are not all possible N-P-K combinations (eg N3P3K3). How did you select these ones?

**Reply:** Thanks for your comment. I search for the corresponding orthogonal table according to the number of factors (3 factors), the number of levels (3 levels) of factors and whether there is interaction, and then select some representative points from the comprehensive test based on the orthogonality of the orthogonal table to carry out the test, so as to achieve the results equivalent to a large number of comprehensive tests with the least number of tests.

**Comment:** Page 7, Table 2: You have not explained what is orthogonal test design. Provide a relative reference.

**Reply:** Thanks for your comment. Orthogonal experimental design is a method of experimental design to study multiple factors and levels. According to the orthogonality, select some representative points which have the characteristics of uniform dispersion, integrity and comparability from the overall test. Orthogonal experimental design is the main method of fractional factorial design. When there are 3 or more factors involved in the test, and there may be interaction between the factors, the test workload will become large, or even difficult to implement. To solve this problem, orthogonal experimental design is undoubtedly a better choice. The main tool of orthogonal test design is orthogonal table. The experimenter can find the corresponding orthogonal table according to the number of factors in the test, the level of factors and whether there is an interaction, and then select some representative points from the comprehensive test based on the orthogonality of the orthogonal table to carry out the test, which can achieve the results equivalent to a large number of comprehensive tests with the least number of tests, Therefore, orthogonal table design is an efficient, fast and economical multi factor test design method.

For example, conduct a three factor experiment with three levels for each factor. If the overall test is conducted, 3 * 3=27 times shall be conducted. If a part of the 27 tests are selected, A and B are usually fixed on the A1 and B1 levels respectively, matching with the three levels of C, A1B1C1, A1B1C2, A1B1C3. After these three tests, if A1B1C3 is the best, set the level of C3, let A1 and C3 be fixed, and then match with the three levels of factor B, A1B2C3 and A1B3C3. After these two tests, if A1B2C3 is optimal, determine the two levels of B2 and C3, and then conduct two tests of A2B2C3 and A3B2C3, and then compare them with each other. If A3B2C3 is optimal, it can be asserted that A3B2C3 is the best level combination we want to select. In this way, the best level combination was selected after only 7 tests. We found that these test results were distributed in one corner of the cube, which was not representative, so the test level combination selected according to the above method was not really the best combination.

Figure 1 Schematic Diagram of Orthogonal Test Design

If the orthogonal test design is carried out and the orthogonal table is used to arrange the test, nine tests are required for the test of three factors and three levels with “ Δ” marked in Figure 1. If each plane represents a level and there are nine planes, you can see that there are three on each plane“ Δ” Point. There is one “ Δ” points on each straight line of the cube and these points are evenly distributed, so the nine tests are very representative and can reflect the results of the comprehensive test comprehensively, which is the unique balanced dispersion characteristic of orthogonal experimental design. We use this feature to reasonably design and arrange tests so as to find the best combination of levels by minimizing the number of tests.

**Comment:** Page 7, Table 2: I propose the following legend: Table 2. Treatments of N, P and K with their dosages on blueberry plants.

**Reply:** Thanks for your suggestion. Table 2 legend has been modified.

**Comment:** Page 7, Table 2: It would be better to present the doses of nutrients (N, P2O5, K2O) not the form of fertilizers (ammonium sulfate etc)

**Reply:** Thanks for your suggestion. Table 2 has been modified.

**Comment:** Page 7, line 126: The Folin-Ciocalteu…method was used to establish …. (mg/g FW)[23].

**Reply:** Thanks for your suggestion. Relevant contents have been modified in lines 126-127 of the manuscript. .

**Comment:** Page 7, line 128: Replace ‘modified’ with ‘determined’

**Reply:** Thanks for your suggestion. It has been revised in line 128 of the manuscript.

**Comment:** Page 8, line 133: Refer the type and model of the refractometer device.

**Reply:** Thanks for your suggestion. The type and model of refractometer device have been added in line 133 of the manuscript.

**Comment:** Page 8, line 136: …SPSS 18 software programs were used…

**Reply:** Thanks for your suggestion. It has been revised in line 136 of the manuscript.

**Comment:** Page 8, line 137: ANOVA and least significant difference (LSD) were applied to compare

**Reply:** Thanks for your suggestion. Relevant contents have been modified in lines 137-138 of the manuscript.

**Comment:** Page 8, line 138: Delete ‘calculate’

**Reply:** Thanks for your suggestion. The word has been deleted from line 138 of the manuscript.

**Comment:** Page 8, line 139: levels was calculated and the principal …evaluation was used.

**Reply:** Thanks for your suggestion. Relevant contents have been modified in lines 139-140 of the manuscript.

**Comment:** Page 8, line 141: Results and Discussion

**Reply:** Thanks for your suggestion. It has been revised in line 141 of the manuscript.

**Comment:** Page 8, line 145: effects were observed in F2 and F6 treatments, which gave 8.67% higher values than ...

**Reply:** Thanks for your suggestion. Relevant contents have been modified in lines 146-147 of the manuscript.

**Comment:** Page 8, line 149: best fertilizer combinations affecting the fruit weight and yield…

**Reply:** Thanks for your suggestion. Relevant contents have been modified in lines 150-151 of the manuscript.

**Comment:** Page 9, line 152: P>K>N, respectively…

**Reply:** Thanks for your suggestion. It has been revised in line 153 of the manuscript.

**Comment:** Page 9, line 154: Delete ‘This is the’

**Reply:** Thanks for your suggestion. Relevant contents have been deleted in line 154 of the manuscript.

**Comment:** Page 9, line 156: The Table 3 repeats data that has been already

**Reply:** Thanks for your suggestion. The repeated data are listed to facilitate range analysis.

**Comment:** Page 9-10, line 156: In the Table 3, column ‘Code’, there is a Chinese character.

**Reply:** Thanks for your suggestion. We are sorry for this error and have revised Table 3 in the manuscript.

**Comment:** Page 10, line 160, 166, 168: sugar: acid

**Reply:** Thanks for your suggestion. Relevant contents in the revised manuscript.

**Comment:** Page 10, line 172: titratable acidity of other

**Reply:** Thanks for your suggestion. It has been revised in line 173 of the manuscript.

**Comment:** Page 10, line 173: but there is no

**Reply:** Thanks for your suggestion. It has been revised in line 174 of the manuscript.

**Comment:** Page 11, line 174: effect was that of treatment F9 (17.07% lower than CK)(Fig 2. G)

**Reply:** Thanks for your suggestion. Relevant contents have been modified in lines 174-175 of the manuscript.

**Comment:** Page 11, line 176-177: F2 was significantly higher (36.36%) than CK…

**Reply:** Thanks for your suggestion. Relevant contents have been modified in lines 177-178 of the manuscript.

**Comment:** Page 11, line 180: is significantly higher (20.59%) than CK…

**Reply:** Thanks for your suggestion. It has been revised in line 181 of the manuscript.

**Comment:** Page 11, line 187: on the above-mentioned parameters of blueberry fruit is…

**Reply:** Thanks for your suggestion. It has been revised in line 187 of the manuscript.

**Comment:** Page 11, line 189: ,respectively. According…

**Reply:** Thanks for your suggestion. It has been revised in line 188 of the manuscript.

**Comment:** Page 12, line 194: Effect of different fertilization formulas on blueberry fruit quality.

**Reply:** Thanks for your suggestion. It has been revised in line 192 of the manuscript.

**Comment:** Page 12, line 196: Table 4. Orthogonal test on …

**Reply:** Thanks for your suggestion. Legend of Table 4 in the revised manuscript.

**Comment:** Page 13, line 199: yield and berry quality of blueberry.

**Reply:** Thanks for your suggestion. It has been revised in line 196 of the manuscript.

**Comment:** Page 13, line 200: The yield and berry quality of blueberry were evaluated…

**Reply:** Thanks for your suggestion. It has been revised in line 197 of the manuscript.

**Comment:** Page 14, line 203-204: Limit to two decimal digits

**Reply:** Thanks for your suggestion. Relevant contents have been modified in lines 200-201 of the manuscript.

**Comment:** Page 14, line 212: treatments F2…

**Reply:** Thanks for your suggestion. It has been revised in line 211 of the manuscript.

**Comment:** Page 14, line 213: were ranked second.

**Reply:** Thanks for your suggestion. It has been revised in line 212 of the manuscript.

**Comment:** Page 14, line 215, 216, 218: and berry quality

**Reply:** Thanks for your suggestion. Relevant contents in the revised manuscript.

**Comment:** Page 14, line 217: Delete ‘which was also F2’

**Reply:** Thanks for your suggestion. Relevant contents have been deleted in the manuscript.

**Comment:** Page 15, table 5: Titratable acidity

**Reply:** Thanks for your suggestion. Relevant contents of Table 5 in the manuscript have been modified.

**Comment:** Page 16, line 223: yield and fruit quality of trees. Studies have shown that these nutrients and their…

**Reply:** Thanks for your suggestion. Relevant contents have been modified in lines 225-226 of the manuscript.

**Comment:** Page 16, line 226: improve fruit quality

**Reply:** Thanks for your suggestion. It has been revised in line 228 of the manuscript.

**Comment:** Page 16, line 233-234: it is not clear what you mean. Was the level of soil K in the region low?

**Reply:** Thanks for your comment. Referring to the nutrient grading standard of the second soil survey in China, the following table shows that the soil potassium content in this area is 2.8g/kg, which is at a very low level.

**Comment:** Page 16, line 235-236: In accordance, we found that …

**Reply:** Thanks for your suggestion. Relevant contents have been modified in lines 236-237 of the manuscript.

**Comment:** Page 16, line 237: titratable acidity in blueberry fruit. There was also significant improvement of the flavonoid…

**Reply:** Thanks for your suggestion. Relevant contents have been modified in lines 238-239 of the manuscript.

**Comment:** Page 16, line 238 and 240: sugar: acid

**Reply:** Thanks for your suggestion. Relevant contents in the revised manuscript.

**Comment:** Page 16, line 239: Delete ‘on’

**Reply:** Thanks for your suggestion. The word has been deleted from the manuscript.

**Comment:** Page 16, line 240: fertilizer on the soluble sugar content, Vc and sugar: acid ratio.

**Reply:** Thanks for your suggestion. It has been revised in line 241 of the manuscript.

**Comment:** Page 16, line 241: Since K can participate in…

**Reply:** Thanks for your suggestion. It has been revised in line 241 of the manuscript.

**Comment:** Page 17, line 242: sugar, it increases the sugar…promotes …

**Reply:** Thanks for your suggestion. Relevant contents have been modified in lines 242 of the manuscript.

**Comment:** Page 17, line 246: Replace ‘extremely’ with ‘highly’

**Reply:** Thanks for your suggestion. It has been revised in line 246 of the manuscript.

**Comment:** Page 17, line 246: It was shown that the

**Reply:** Thanks for your suggestion. Relevant contents have been modified in lines 246 of the manuscript.

**Comment:** Page 17, line 248: It was shown that the

**Reply:** Thanks for your suggestion. It has been revised in line 248 of the manuscript.

**Comment:** Page 17, line 252: and applying more (but not excessive) P fertilizer …

**Reply:** Thanks for your suggestion. Relevant contents have been modified in lines 251-252 of the manuscript.

**Comment:** Page 17, line 252-253: Delete ‘but it does not mean adding excessive phosphorus fertilizer.’

**Reply:** Thanks for your suggestion. This sentence has been deleted from the manuscript.

**Comment:** Page 17, line 256: low demands for

**Reply:** Thanks for your suggestion. It has been revised in line 254 of the manuscript.

**Comment:** Page 17, line 259: and berry quality of blueberry, it is necessary to determine and apply the appropriate…

**Reply:** Thanks for your suggestion. Relevant contents have been modified in lines 257-258 of the manuscript.

**Comment:** Page 18, line 263: fertilization formula

**Reply:** Thanks for your suggestion. It has been revised in line 261 of the manuscript.

**Comment:** Page 18, line 264: but all treatments could improve them by …

**Reply:** Thanks for your suggestion. It has been revised in line 262 of the manuscript.

**Comment:** Page 18, line 265-266: Moreover, heavy fertilization does not mean …

**Reply:** Thanks for your suggestion. It has been revised in line 264 of the manuscript.

**Comment:** Page 18, line 267: Replace ‘obtain’ with ‘result in’

**Reply:** Thanks for your suggestion. It has been revised in line 265 of the manuscript.

**Comment:** Page 18, line 271-275: Replace ‘that is, the best … potassium sulfate’ with ‘that is, 100, 25 and 25 g/plant of N, P2O5 and K2O, respectively.’

**Reply:** Thanks for your suggestion. Relevant contents have been modified in lines 269-270 of the manuscript.

**Comment:** Page 18, line 277-281: It is not clear what you mean here. Rewrite it better.

**Reply:** Thanks for your suggestion. The passage in line 271-275 of the manuscript has been rewritten.

**Comment:** Page 19, references: All latin names of plants must be written in italics.

**Reply:** Thanks for your suggestion. All latin names of plants in the references have been modified to be written in italics.
