# Supplementary material for: Effects of nitrogen, phosphorus and potassium formula fertilization on the yield and berry quality of blueberry
Source: PLoS One. 2023 Mar 16;18(3):e0283137. doi: 10.1371/journal.pone.0283137 (PMC10019695; doi:10.1371/journal.pone.0283137)
Supplement: Attachment — Submitted filename: rebuttal letter.docx [file pone.0283137.s002.docx]

**Detailed Response to Reviewers**

**Manuscript Number:** PONE-D-22-30089

**Title:** Effects of nitrogen, phosphorus and potassium formula fertilization on the yield and berry quality of blueberry

**Authors:** Xinyu Zhang^1^,Shuangshuang Li^2^, Delu Wang^1*^,Xiaoli An^1^,Zejun Song^1^, Yunzheng Zhu^1^,Yi Tan^1^,Xiaolan Guo^3^

Dear Editor: Sushanta Kumar Naik and Dear reviewers:

Thank you very much for your useful comments and professional advice on our manuscript. We wish to give a sincere gratitude to referees for reviewing our paper carefully. These opinions help to improve academic rigor of our article, and we apologize for any inconveniences caused by these errors. We have modified the manuscript accordingly, and the response to the referees’ comments are listed point by point below:

**Reviewer #1:**

**Comment 1:** Soil pH is in acidic range, so significant amount of the applied phosphorus may be fixed in soil, so, whether, it is good to apply P fertilizer directly without any soil amendments.

**Reply 1:** Thanks for your comment. Although a large amount of phosphorus is fixed in the soil under acidic conditions, it is not the best to apply phosphorus fertilizer directly. Because applying only one kind of fertilizer will aggravate the imbalance of soil nutrients. Moreover, a large number of studies have shown that compared with the single application of nitrogen, phosphorus and potassium, the combined application can significantly improve the fertilizer utilization rate.

**Comment 2:** It is still not mentioned in the manuscript that, what is the basis of selection of three levels of N, P and K, How you get the level 1.

**Reply 2:** Thanks for your comment. Select the three factors of N fertilizer, P fertilizer and K fertilizer, and set three levels for each factor according to Guo et al. [22], which has been added to line 3 on page 3 of the manuscript.

**Comment 3:** Selection of recommendation should be based on the B: C ratio or some other important aspects. Which is not mentioned clearly?

**Reply 3:** Thanks for your suggestion. It is calculated that the benefit-cost ratio of treating F2 and F4 is 16.13 and 15.87, respectively, so F2 processing is more recommended, and the relevant content has been added to lines 16-17 on page 6 of the manuscript.

**Comment 4:** Presentation of results in figures and tables are complicated, so, if possible convert it in simple form.

**Reply 4:** Thanks for your suggestion. But I can't find a better way to show the results. If possible, I hope you can give me some advice.

**Reviewer #2:** There are yet some missing data as follows: Materials and methods

**Comment 1:** If available, provide the mean nutrient status of tested plants before fertilizer treatments took place.If available, provide data about the fertilizers applied the year before experimentation.What were the plantation distances and plantation density (number of trees/ha) of the tested orchard?What about canopy training system and mean height of the tested trees?

**Reply 1:** Thanks for your suggestion. We regret that we did not provide enough complete material information in the materials and methods section. First of all, the plantation distances and plantation density (number of trees/ha) of the tested orchard are 1.5m and 4444 trees/ha respectively, which have been added to lines 4-5 on page 3 of the manuscript. Secondly, as a response to the reviewers, we regret the lack of detailed data on the mean nutrient status of tested plants before fertilizer treatments took place, the data of the fertilizers applied the year before experimentation, the canopy training system and mean height of the tested trees. This is a mistake in our research. We will be more cautious in future research.

**Comment 2:** Page 2, line 11: Replace ‘plants’ with ‘species’.

**Reply 2:** Thanks for your suggestion. The relevant content has been revised in line 10 on page 2 of the manuscript.

**Comment 3:** Page 2, bottom line: Replace ‘was’ with ‘were’.

**Reply 3:** Thanks for your suggestion. The last line on page 2 of the manuscript has been revised.

**Comment 4:** Discussion, line 11: Delete the word ‘fertilizer’.

**Reply 4:** Thanks for your suggestion. Relevant contents are modified in line 11 of the discussion part of the manuscript.

**Comment 5:** Conclusion, first line: …formula on blueberry fruit …

**Reply 5:** Thanks for your suggestion. Relevant contents are revised in the first line of the conclusion of the manuscript.

**Comment 6:** Conclusion, bottom line: …of increasing N and K, and stabilizing P.

**Reply 6:** Thanks for your suggestion. The last line of the conclusion of the manuscript has been revised.
